# Two new species of Gingers (Zingiberaceae) from Myanmar

**DOI:** 10.3897/phytokeys.13.2670

**Published:** 2012-06-07

**Authors:** Vinita Gowda, W. John Kress, Thet Htun

**Affiliations:** 1 Dept. of Botany, MRC-166, P. O Box 37012, Smithsonian Institution, Washington, D.C. 20013-0712, USA; 2 Ministry of Forestry, Department of Forestry, Office No. 28, Nay Pyi Taw, Union of Myanmar

**Keywords:** *Curcuma*, endemic, *Globba*, Myanmar, new species, taxonomy, Zingiberaceae

## Abstract

Two new species of gingers (Zingiberaceae), *Globba sherwoodiana* W.J. Kress & V. Gowda **sp. nov.**, and *Curcuma arracanensis* W.J. Kress & V. Gowda **sp. nov.**, from Myanmar are described. The new species of *Globba* is currently only known in cultivation and is commonly grown and sold in markets in Myanmar. In contrast *Curcuma arracanensis* has been collected from a single restricted region in the cloud forests of the Rakhine Yoma above the Bay of Bengal in western Myanmar. Three-locus DNA barcodes were generated as aids for the identification of the two new species.

## Introduction

A recent surge of interest in the taxonomy and classification of the family Zingiberaceae (Kress et al. 2002, 2007) as well as significant efforts at field exploration in Southeast Asia have resulted in the discovery and description of a plethora of new species and genera of gingers (e.g., Kress et al. 2010, Leong-Škorničková et al. 2011). The abundance of gingers in the understory of evergreen and monsoonal forests and the common uses of gingers as spices, medicines, and ornamentals has led to new discoveries in both natural environments (Kress et al. 2010) as well as the marketplace (Kress and Larsen 2001, Kress and Htun 2003). Here we describe two new species from Myanmar, one found on the top of a ridge in pristine cloud forest and the other as a common offering in Buddhist ceremonies and temples.

## Methods

The morphology of both new species was analyzed using herbarium material and living plants cultivated in the Department of Botany Research Greenhouses at the Smithsonian (USBRG). Detailed morphological measurements were made using digital calipers and a calibrated eye piece under a dissecting microscope. Herbarium specimens to serve as types were taken from plants grown in cultivation when required (see Taxonomic Treatment).

DNA extraction, amplification and sequencing were carried out for three barcoding regions *rbcL*, *matK*, and the *trnH-psbA* spacer region using published primers under standard conditions (see Kress and Erickson 2007). Genbank accession numbers for all the sequences are listed in Table 1.

**Table 1.** Voucher information and Genbank accession numbers for *Globba sherwoodiana* and *Curcuma arracanensis*.

**Species**	**Gene region**	**Genbank accession number**	**Voucher (Herbarium location)**
*Globba sherwoodiana*	*rbcL*	1504642	WJK 00-6669 (US)
	*matK*	JQ480153	WJK 00-6669 (US)
	*trnH-psbA*	1504642	WJK 00-6669 (US)
*Curcuma arracanensis*	*rbcL*	1504641	WJK 03-061 (US)
	*matK*	JQ480152	WJK 03-061 (US)
	*trnH-psbA*	JX082290	WJK 03-061 (US)

## Taxonomic treatment

### Globba sherwoodiana

W.J.Kress & V.Gowda sp. nov.

urn:lsid:ipni.org:names:77119952-1

http://species-id.net/wiki/Globba_sherwoodiana

Fig. 1 Plate 1.

#### Diagnosis.

A new species in the genus *Globba* Section *Globba* differing from other species in this section, such as *Globba laeta* K.Larsen, *Globba marantina* L., and *Globba winitii* C.H.Wright by the combination of white, sharply reflexed and imbricate inflorescence bracts and soft, glabrous, bright green leaves.

#### Type.

**Myanmar**: Mandalay Division: Pyin Oo Lwin, National Botanical Gardens, 69 km from Mandalay, 1000 m, 21°59'31"N, 96°28'09"E, *W. J. Kress, Thet Htun, and M. Bordelon s.n.* (only living plant collected for cultivation); Plants of this living collection cultivated at the Smithsonian Botany Research Greenhouses as USBRG 1997–141, 29 September 2011, *W. J. Kress, V. Gowda & M. Bordelon 11–8809* (holotype: US! [US 3635561, barcode 00940954; isotypes RAF!, E!).

#### Description.

Small perennial herbs from 38–45 cm in height to the top of uppermost leaf sheath. Rhizomes compact, non-tuberulous, white with a light orange center internally. Leafy shoots densely clumped, 6 to 8-leaved, stems bright green in color, sparsely hirsute. Basal sheaths 5–7 × 1–2 cm, sparsely hirsute. Plane of distichy perpendicular to rhizome. Leaves glabrous and soft, only midvein of the ventral surface pubescent, lamina 17–20 × 5–8 cm elliptic bright green adaxially and pale green abaxially, margin entire, base attenuate, apex acuminate; petiole 0.5–0.7 × 0.3–0.4 cm, sparsely hirsute, green. Ligule small, 2–3 mm in length, hirsutulous, emarginate not becoming papery. Inflorescence terminal on leafy shoots, pendent 11–15 cm; peduncle 2.5–4 × 0.35–0.5 cm, hirsute, pale green; rachis hirsute, straight, visible; inflorescence bracts 25–30 per inflorescence, bracts decreasing in size from base to the tip, median bract 2.5–3.5 cm × 1.5–1.8 cm, spirally arranged, imbricate and widely separated, sharply reflexed105–107°from vertical axis, glabrous, white. Cincinni ca. 1.4–2 cm long to the first flower, originating under the inflorescence bract, 2–8 mm apart on the rachis, one per bract containing 2–3 flowers; flowers maturing from base to apex of inflorescence; bracteoles tubular, basal and largest bracteole 2.6–3 × 3.5–5 mm, glabrous, light green. Flowers conspicuous; calyx tubular 4.5–5 mm long, reflexed at ca. 3–5mm from base, tri-lobed, yellow green; corolla tube 1.1–1.4 cm long, sparsely puberulous, with lobes reflexed; lobes cucullate, ca. 4–6 mm × 2–3 mm; lateral staminodes 7–9 × 2.3–2.6 mm in length, elliptic, glabrous, orange; labellum 6.2–7.6 × 2.2–2.4 mm, triangular, bi-lobed, glabrous, orange with deep orange center; fertile stamen with filament 1.6–2.6 cm long, orange, glabrous, anther 2.2–2.6 mm long, thecae elliptic with four appendages, glabrous, crest not extended beyond thecae; style held in the ventral furrow of the filament; stigma cup-shaped, pubescent with ciliate margin; ovary uni-locular, 1.2–2 × 1.6–2.7 mm, glabrous, white, with parietal placentation. Epigynous (stylodial) nectaries 2, linear septal, 3–4 mm long, light orange. Fruit and seeds unknown. Bulbils not observed.

**Figure 1.**
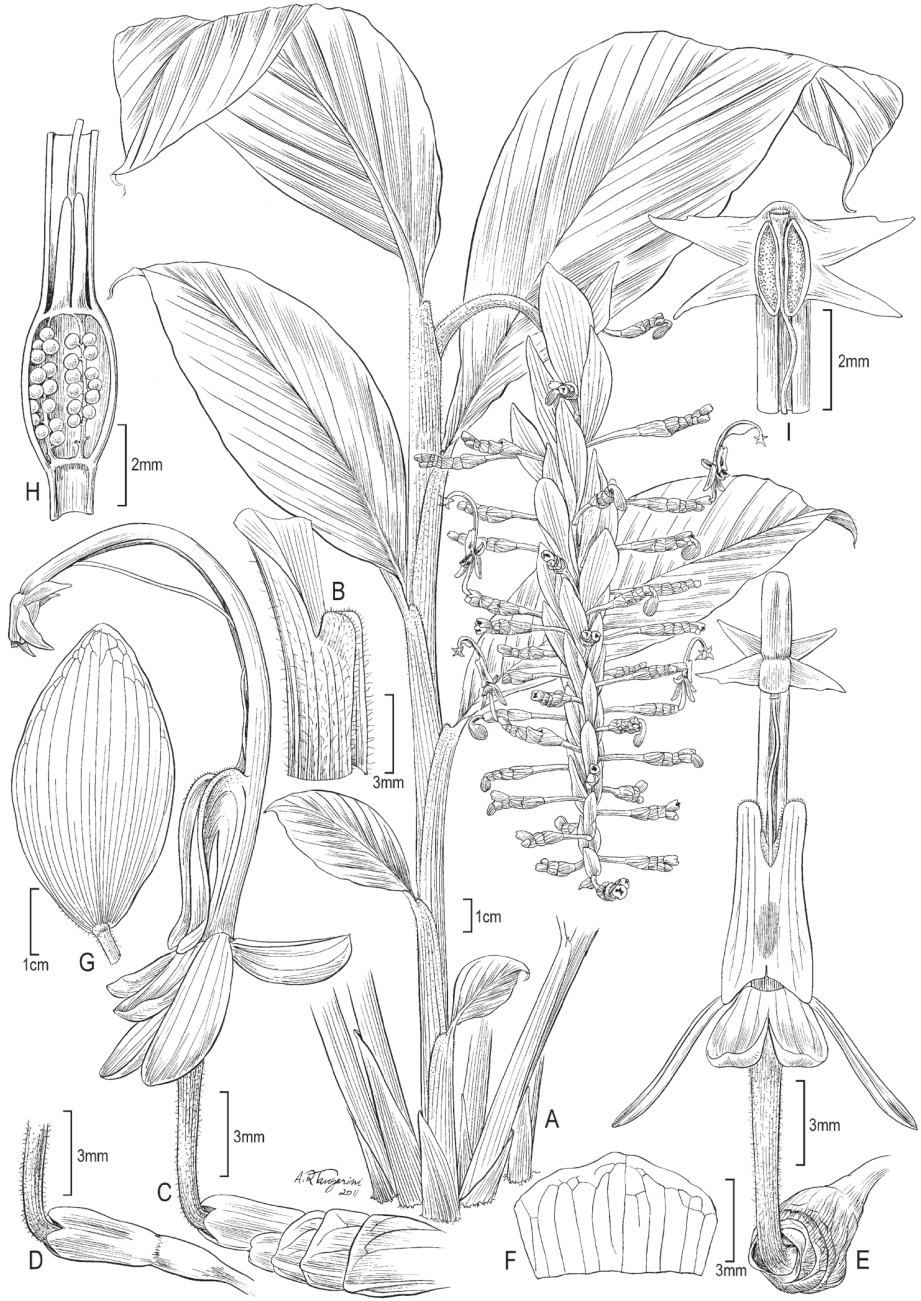
*Globba sherwoodiana* W.J.Kress & V.Gowda. **A** Habit of flowering plant **B** Apical part of leaf sheath showing ligule **C** Flower, lateral view showing bracteoles **D** Calyx with floral tube **E** Flower, frontal view **F** Bracteole **G** Inflorescence bract **H** Base of flower, cut-away view showing style, epigynous nectaries and placentation **I** Anthers with four appendages, style held in the ventral furrow of the filament with a cup-shaped stigma. WJK 11-8809 (US).

#### Distribution.

At present this species is only known in cultivation in Myanmar where it is commonly sold in the markets and used as an offering in Buddhist ceremonies. However, it is suspected that wild populations are present in the border region between Myanmar and Thailand.

#### Ecology.

The closest relatives of this species (see relationships) inhabit the understory of monsoon forests. We suspect that the same is true for *Globba sherwoodiana*.

#### Etymology.

This splendid and magnificent species of *Globba* is named in honor of Dr. Shirley Sherwood, a great lover of plants and a patron of contemporary botanical art. Her support of botanical research in Myanmar is greatly appreciated.

#### Common name.

The local name for this species, “Padein Gno,” means “weeping goldsmith” in the Burmese language. It is said that local goldsmiths in Myanmar weep when they see this flower because no matter how hard they try, they are unable to capture in gold metal the beauty and simplicity of the individual parts of the flower (see Kress 2009).

#### Conservation status.

Because this species is commonly cultivated it is in no danger or threat of extinction. However, the lack of known natural populations may suggest that this cultivated species may have low levels of genetic diversity. Genetic studies will provide a better understanding of the conservation status of this species.

#### Relationships.

*Globba sherwoodiana* was included in the molecular phylogenetic analysis of Williams et al. (labeled as *Globba* “magnifica”; 2004). Using DNA sequence data from *matK* and ITS, they showed that this species is a member of Sect. *Globba laeta* K.Larsen, *Globba bulbifera* Roxb., *Globba schomburgkii* Hook. f., *Globba globulifera* Gagnep., *Globba adhaerens* Gagnep., *Globba marantina* L., and *Globba winitii* C.H.Wright. Several species in this section have large conspicuous inflorescence bracts similar to those characterizing *Globba sherwoodiana*.

#### Other specimens examined.

Known from the type specimen and additional specimens of USBRG 97–141in cultivation; WJK 00–6669; WJK 99–6533.

### Curcuma arracanensis

W.J.Kress & V.Gowda sp. nov.

urn:lsid:ipni.org:names:77119953-1

http://species-id.net/wiki/Curcuma_arracanensis

Fig 2 Plate 2.

#### Diagnosis.

A new species in the genus *Curcuma* differing from other members of the genus known from Myanmar in the inflorescence borne well above the surface of the ground at the apex of a leafy shoot, deep maroon inflorescence bracts, and very conspicuous, bright orange flowers.

#### Type.

**Myanmar**: Rakhine State: 57 miles from Taung-gok towards Pyay, steep hillsides of cloud forest in fine scree soil, 795 m, 18°38'15"N, 94°38'97"E, 20 June 2003, *W. J. Kress, Aye Pe, Than Than Htay, Win Win Aung, and M. Bordelon 03–7328*; living collection cultivated at the Smithsonian Botany Research Greenhouses as USBRG 2003–061, 20 June 2003. *W. J. Kress 03–7328* (holotype: US! [US 3572390, barcode 00940953; isotypes RAF!; E!).

####  Description.

Medium herb to 85 to 130 cm in height; rhizomes compact, yellow internally, with numerous white tubers (yellow internally). Leafy shoots loosely clumped, disarticulating during dry season, 3 to 5-leaved, with basal sheaths green with red speckles, glabrous, 20–22 × 4–5 cm. Plane of distichy parallel to rhizome. Leaves 60–70 cm in length, glabrous and coriacious; petiole 19–23 0.7–0.8 cm, glabrous, green with small red speckles, deeply grooved in cross-section, margin entire, smooth; ligule medium-sized, 1.5–3.2 cm in length, bi-lobed, thin and translucent, pale yellow green, glabrous; blade 43–49 × 17–20 cm, narrowly ovate, midrib below green with sparse red speckles, glabrous, base cordate, subequal, apex caudate**,** adaxial surface dark green. Inflorescence terminal on relatively long leafy shoot, erect 19–25 cm in height; peduncle 2–5 cm in length, glabrous, green to deep maroon red; rachis short; inflorescence bracts 25–30 per inflorescence, 2.4–2.9 × 2.5–3.4 cm, spirally arranged and imbricate, each fused at base to adjacent members (“pouched’), 40–50° from vertical axis, glabrous, green basally to deep red maroon distally; no coma. Cincinni one per bract containing 3–4 flowers, maturing from base to apex of inflorescence; bracteoles not tubular, 13–15 × 4–6 mm, translucent, glabrous, white with short red apex. Flowers with tubular calyx, 16–22 mm long, tri-lobed, sparsely hirsute with very short hairs, pale yellow orange; corolla tube 2.8–3.5 cm, orange to pale orange, glabrous, with lobes not reflexed, 19–22 mm in length; lateral staminodes fused to base of filament of fertile anther, 13–17 × 7–10 mm, bluntly acute, glabrous, orange; labellum 18–22 × 15–18 mm, spatulate, shallowly bi-lobed at apex, glabrous, yellow orange with deep orange central stripe and reddish margins, lobes not flared; fertile stamen with filament 7–9 mm long, 4–5 mm wide, glabrous, orange, anther versatile, 10–12 × 4.5 mm, thecae elongate with minute, blunt spurs, glabrous, orange, crest much reduced extended <1 mm beyond thecae; stigma shallowly cup-shaped, ovary tri-locular, 3–5 × 3–4 mm, glabrous, pale yellow or green, placentation axile. Epigynous (stylodial) nectaries 2, rounded, 2.4–2.8 mm long, with scattered minute hairs, pale orange to cream-colored; a collar of short hairs forming nectar chamber on inside of corolla tube 5 mm above apex of ovary. Fruit and seeds unknown.

**Figure 2.**
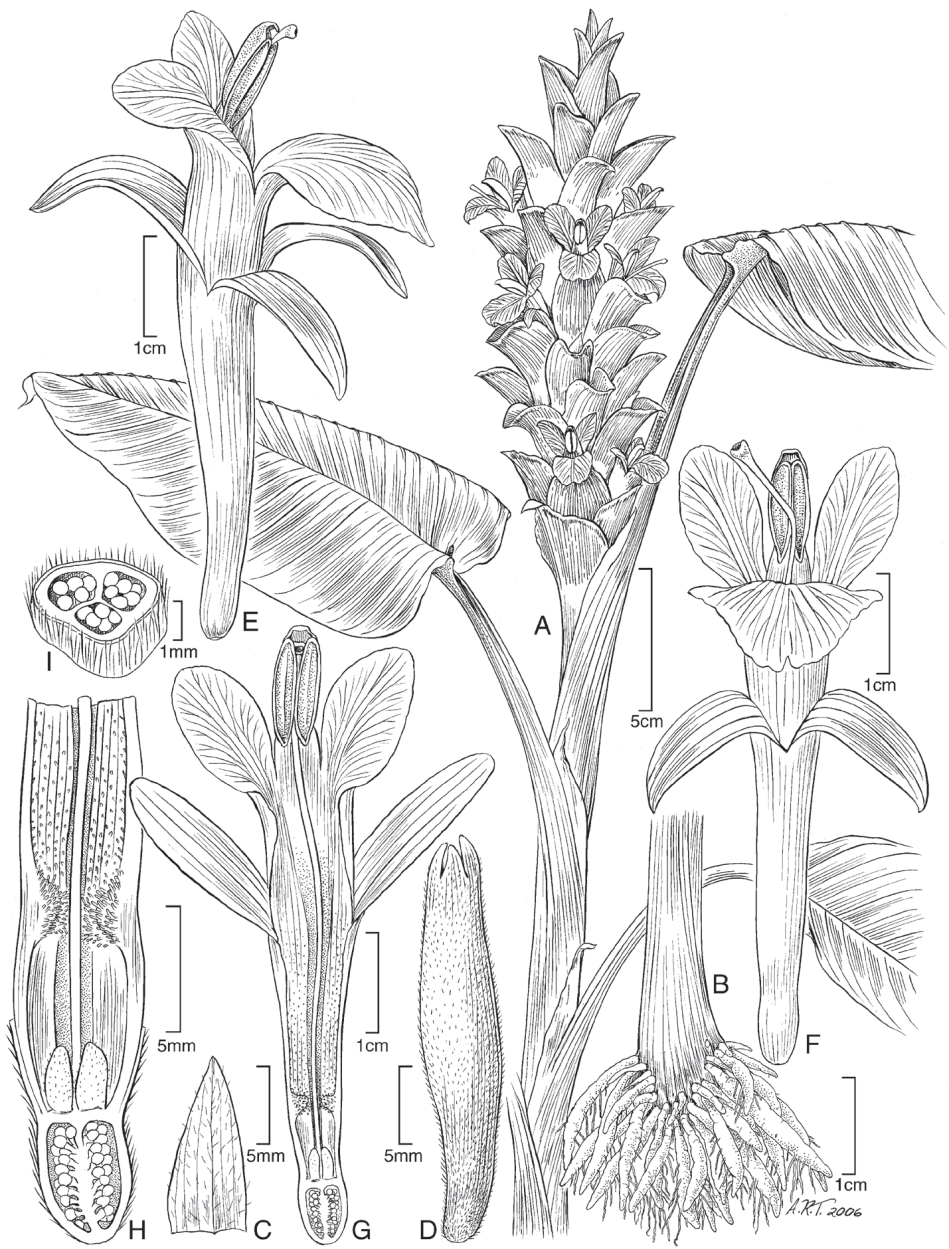
*Curcuma arracanensis* W.J.Kress & V.Gowda. **A** Habit of flowering plant **B** Tuberous rhizome **C** Ligule **D** Bracteole **E** Flower, lateral view **F** Flower, frontal view **G** Flower, cut-away view showing style and anthers **H** Base of flower, cut-away view showing style, epigynous nectaries and placentation **I **Placentation, cross section. WJK 03-7328 (US).

**Plate 1.**
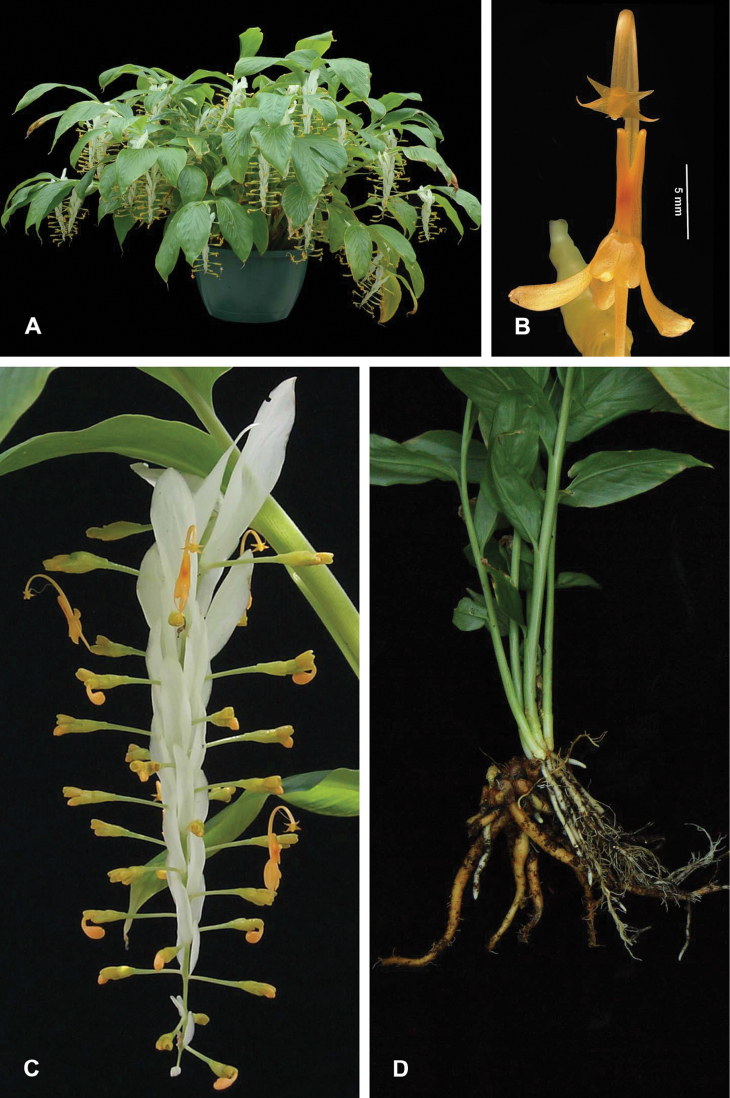
*Globba sherwoodiana* W.J.Kress & V.Gowda. **A** Habit **B** Flower**,** front view showing two lateral staminodes, two petal lobes, labellum, and the four appendages of the anther **C** Inflorescence, lateral view **D** rhizome. WJK 97-141 (USBRG).

**Plate 2.**
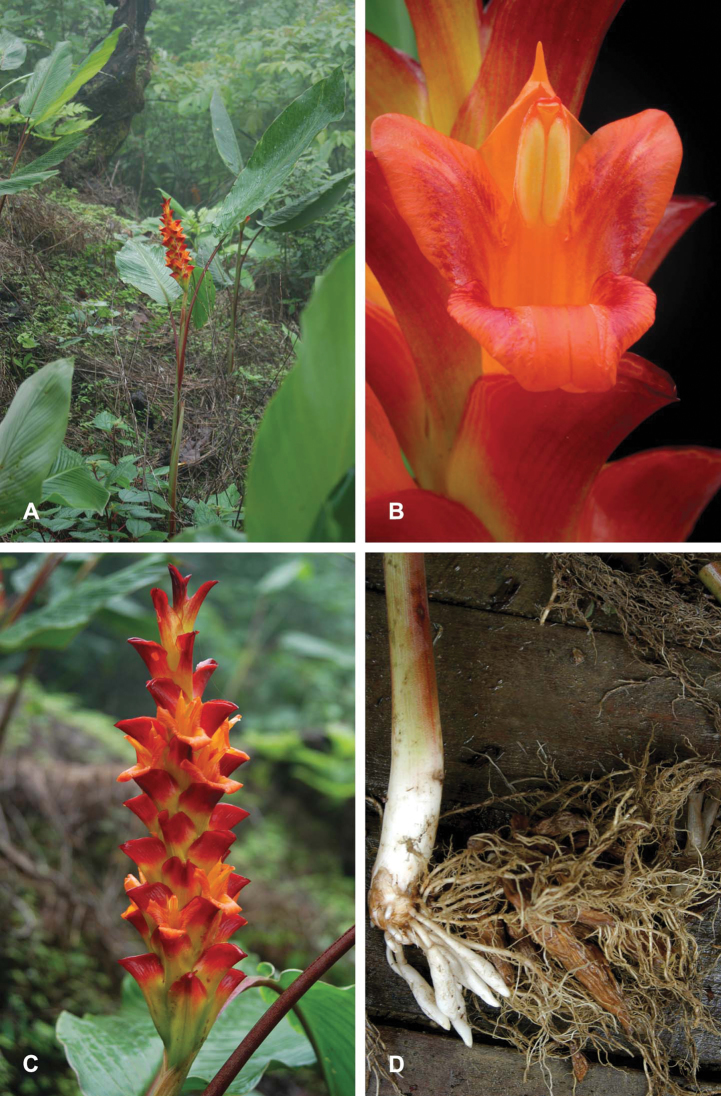
*Curcuma arracanensis* W.J.Kress & V.Gowda. **A** Habit **B** Flower, frontal view **C** Inflorescence **D** Rhizome. WJK 03-7328 (US).

#### Distribution.

This species is known only from the area around the type locality in Rakhine State, Myanmar.

#### Ecology.

Unlike many species of *Curcuma*, which are found in the understory of seasonally dry monsoonal forests, *Curcuma arracanensis* inhabits open areas in evergreen cloud forest.

#### Etymology.

This species is named for the region formerly known as Arrakan, an earlier name for today’s Rakhine State in Myanmar.

#### Common name.

The local name for this species is “Tauk tar phu” in the Burmese language.

#### Other specimens examined.

Known only from the type specimen. Collections measured WJK 03–7328; USBRG 2003–061.

#### Conservation status.

Because this species is only known from a single locality in Myanmar and the habitat in which it is found is steadily declining due to deforestation, we categorize it as critically endangered under criteria B and D following the IUCN guidelines (IUCN Standards and Petitions Subcommittee 2011).

#### Relationships.

The relationship of *Curcuma arracanensis* to other members of the genus is not known at present. The anther shape is similar to that found in *Curcuma petiolata* Roxb (distributed from India to Malaysia including Thailand) and may suggest evolutionary affinities to species allied to this taxon. Further molecular and morphological analyses are needed to determine more precise relationships.

## Supplementary Material

XML Treatment for Globba sherwoodiana

XML Treatment for Curcuma arracanensis
